# Selenium and mercury in the hair of raccoons (*Procyon lotor*) and European wildcats (*Felis s. silvestris*) from Germany and Luxembourg

**DOI:** 10.1007/s10646-019-02120-3

**Published:** 2019-11-16

**Authors:** Danuta Kosik-Bogacka, Natalia Osten-Sacken, Natalia Łanocha-Arendarczyk, Karolina Kot, Bogumiła Pilarczyk, Agnieszka Tomza-Marciniak, Joanna Podlasińska, Mateusz Chmielarz, Mike Heddergott, Alain C. Frantz, Peter Steinbach

**Affiliations:** 1 Independent of Pharmaceutical Botany, Department of Biology and Medical Parasitology, Pomeranian Medical University in Szczecin, Powstanców Wielkopolskich 72, 70-111 Szczecin, Poland; 2 Institute of Veterinary Medicine, Faculty of Biological and Veterinary Sciences, Nicolaus Copernicus University, Gagarina 7, 87-100 Toruń, Poland; 3 Fondation faune-flore, 25 rue Muenster, L-2160 Luxembourg, Luxembourg; 4 Department of Biology and Medical Parasitology, Pomeranian Medical University in Szczecin, Powstanców Wielkopolskich 72, 70-111 Szczecin, Poland; 5 Department of Animal Hygiene and Prophylaxis, West Pomeranian University of Technology, Judyma 6, 71-466 Szczecin, Poland; 6 Department of Ecology, Environmental Management and Protection, West Pomeranian University of Technology, Słowackiego 17, 71-434 Szczecin, Poland; 7 National Museum of Natural History, 25 Rue Münster, 2160 Luxembourg, Luxembourg; 8 University of Göttingen, Faculty of Chemistry, Tammannstraße 4, 37077 Göttingen, Germany

**Keywords:** Hair, Total mercury, Selenium, Raccoon, European wildcat

## Abstract

This study examined the concentration of total mercury (THg) and selenium (Se), as well as the molar ratio of Se:THg in hair samples of terrestrial animals. THg and Se concentrations were measured from the hair of raccoons (*Procyon lotor*) and European wildcats (*Felis s. silvestris*) from Germany and Luxembourg. Median THg concentrations in hair from raccoons and wildcats were 0.369 and 0.273 mg kg^−1^ dry weight (dw), respectively. Se concentrations were higher in the hair of raccoons than of wildcats (0.851 and 0.641 mg kg^−1^ dw, respectively). Total mercury concentration in hair of raccoons from Luxembourg was almost 5× higher that found in hair of raccoons from Germany; however, Se concentration was similar. Thus, molar ratio of Se:THg was ~4× higher in the hair of raccoons from Germany than those from Luxembourg. Significant negative correlation was found between THg concentration and Se:THg molar ratio in both wildcats and raccoons.

## Introduction

Mercury (Hg) is a chemical element with well-established toxic effects on humans and other terrestrial mammals, reaches its highest concentrations in predatory fish, piscivorous birds, and mammals (Eisler 2000; Scheuhammer et al. 2007; Wiener et al. 2003). Terrestrial vertebrates can absorb Hg through their skin, respiratory, and digestive systems (Graeme and Pollack 1998). Mercury accumulates in internal organs, especially the brain, causing neurotoxicity and impairing development (Castoldi et al. 2001; Davis et al. 1994; Graeme and Pollack 1998). Selenium (Se) is an essential micronutrient for humans and animals (Fordyce 2013; Mao et al. 2016; Thomson 2004). It has numerous important biological functions, serving as the active center of selenoenzymes and selenoproteins, playing an integral role in energy metabolism and gene expression, and functioning in multiple antioxidant, immunoregulatory, and antagonistic roles (Izquierdo et al. 2010; Roman et al. 2014). Selenium has long been recognized for its potential in reducing the toxicity of Hg compounds and has been postulated as a core element in the in vivo demethylation pathway of methylmercury (Bjørklund et al. 2017; Khan and Wang 2010). In animals, interactions between Hg and Se depend mainly on the chemical form and concentration of both elements in the environment duration of exposure, and the species in question (Bjerregaard et al. 2011; Cuvin-Aralar and Furness 1991). A possible pathway for the in vivo demethylation of methylmercury (MeHg) involving Se compounds has been observed, producing the Hg-Se complex as its final degradation product (Khan and Wang 2010). The Hg-Se complex has been observed in the liver, kidney, and brain of marine mammals indicating that this pathway may be active (Lailson-Brito et al. 2012). The Hg:Se molar ratio in animal tissues is an intensively investigated parameter in laboratory and field studies (Cuvin-Aralar and Furness 1991; Raymond and Ralston 2004). Although numerous studies indicate that Se protects against Hg toxicity, studies have also documented that Hg exposure reduced the activity of Se-dependent enzymes and suggested a role of Hg–Se interactions in developmental pathophysiology (Ohlendorf and Heinz 2011; Raymond and Ralston 2004). Nonetheless, this ratio is commonly used to evaluate the susceptibility of animals to Hg toxicity.

Most studies have measured Hg and Se concentrations in internal mammalian tissues, such as the liver and kidney, but there exist effective non-invasive methods for bioindication studies utilizing cutaneous tissues, such as hair, fur and wool. It has been shown that total Hg concentration (THg) in hair is highly correlated with THg in blood and is thought to be marker of THg levels in circulation (Castellini et al. 2012; Lieske et al. 2011; Rea et al. 2013).

Similar relationships are observed in the case of Se. For this reason, some researchers indicate that Se concentration in animal hair may be successfully used to diagnose both deficiency and a high level of this elements in the organism (Clark et al. 1989, Pilarczyk et al. 2019). In the available literature there are a lot of works indicating that hair is useful non-invasive matrice for Se biomonitoring in animal and human body. For example, Górski et al. (2018) found that increase oral intake of Se caused a significant increase (*p* ≤ 0.05) in the Se concentration in the hair of animals. In turn, Cho and Yang (2018), based on their observation, suggested that hair better reflects Se status in organism than serum and therefore they recommend use of hair, as one of matrixes, to full analysis of selenium status. In addition, significant correlations between Se and Hg in hair was reported (Soares de Campos et al. 2002).

Many medium sized mammals widely distributed in forest, agricultural and urban landscapes of the central Europe, with same species introduced into areas beyond their natural occurrence (Kalisińska 2019). Raccoons *Procyon lotor* (Linnaeus, 1758) are bioindicators of environmental contamination, including THg, due to their omnivorous diets and dependence on both aquatic and terrestrial systems for food, making them susceptible to THg biomagnification from consumption of contaminated lower-trophic-level organisms (Bigler et al. 1975; Herbert and Peterle 1990; Lord et al. 2002). The raccoon is a Central and North American carnivore that was introduced into Germany around 70 years ago (Stubbe 1993). The role of the European wildcat (*Felis silvestris* Schreber, 1777), feeds animal prey, in bioindication has not yet been researched. The distribution area of the European wildcat, a medium-sized carnivore is composed of disconnected populations extending from the Iberian Peninsula over southern and central Europe into eastern Europe (Herrmann et al. 2007; Klar et al. 2008). The wildcat is one of the most endangered carnivore species in Europe. The population density declined and the distribution area became fragmented over the last century due loss of habitat, illegal hunting and road kills (Sahl and Artois 1994). The wildcat is a solitary and territorial species and home areas are quite big, range from 175 ha to more than 2000 ha, higher for males (Biró et al. 2004). Fragmentation of the natural habitat can influence migration and possibilities of finding new areas for young individuals. Some treat for young cats are also larger predators, such as foxes, wolves, other cats, and large birds of prey, such as owls and hawks, however adults are secretive and agile and can protect themselves from animals larger than themselves (Nowak 1997). Another treat for wild living populations is mixing with domestic cats and wildcat is facing similar threats across its range in mainland Europe (Pierpaoli et al. 2003). Such genetic mixing may result in individuals that are less well adapted (Rhymer and Simberloff 1996).

The size of the animal population depends on their reproductive success, and it depends on many factors, including exposure to harmful compounds or mineral deficiencies. Excessive Hg and Se concentration in animals results in disturbances in reproduction. In case of Hg, the negative relationship between Hg concentrations and the propensity and ability of adult females to reproduce was reported (Beau et al. 2019). These disorders are explained by endocrine disruptive properties of Hg. In turn Silva et al. (2019) reported that even environmental levels of Hg affects spermatozoa function and fertility capacity in sperm. Also, Se deficiency is related to the problem of reproduction in both males (Ahsan et al. 2014) and females (Qazi et al. 2018).

Considering that Se is capable of metabolic interaction with Hg, it seems to be interesting to assess molar concentrations of THg in relation to Se concentration in raccoons (omnivores) and wildcats (carnivores). Though well-studied in piscivorous animals, reports on the hair levels of THg and Se and the interactions between them in raccoons and wildcats are currently missing from European literature. Therefore, the aim of the study was to investigate the concentration of THg and Se, and Se:THg molar ratio in hair samples taken from raccoons and wildcats.

## Material and methods

### Areas of study

**Fig. 1:**
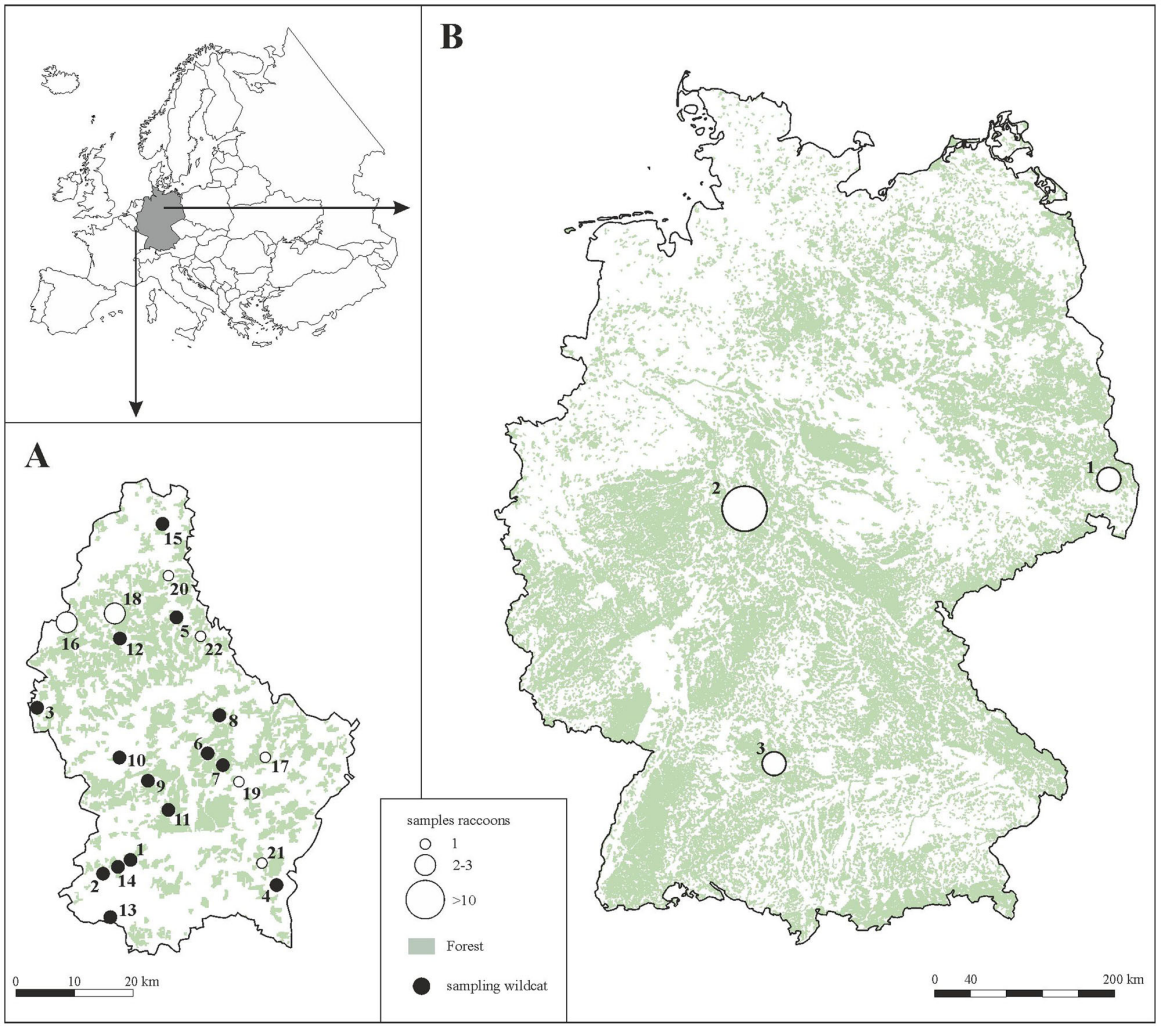
Geographic distribution of the samples from European wildcats (*Felis s. silvestris*) in Luxembourg and raccoons (*Procyon lotor*) in Luxembourg and Germany. **a** Luxembourg. Wildcat (black circle): 1 = Mamer; 2 = Garnich; 3 = Rombach; 4 = Bous; 5 = Hoscheid; 6 = Angelsberg; 7 = Koedange; 8 Medernach; 9 Bour; 10 = Rippweiler; 11 Kopstal; 12 = Buderscheid; 13 = Esch/Alzette; 14 = Dippach; 15 = Rossmillen. Raccoon (white circle): 16 = Tarchamps; 17 = Bech; 18 = Winsler; 19 = Junglister; 20 = Marnach; 21 = Roedt; 22 = Bastendorf. **b** Germany. 1 = Mücka; 2 = City of Kassel; 3 = Geislingen an der Stiege

Samples were collected from three areas in Germany (Hesse, Saxony and Baden-Württemberg) and one area in Luxembourg (Fig. 1). The first of the areas studied corresponded to the city of Kassel (106.78 km^2^), the largest city in the northern state of Hesse (51° 18′N, 9°29′E). The city is located in an extended valley on the flood plain of the Fulda cap River lowercase due to its geographical location in the valley, the Fulda often floods into Kassel and adjacent urban areas. Almost half of this area is cultivated (construction area 35% and transport 13%) and 32% of the area is forest, park and green areas. The expansion of the city in the 20th century saw the settlement and expansion of heavy industry, mechanical and automotive engineering, and the arms industry.

The second investigation area, Mücka, is a small Upper Lusatian municipality in the Saxony district of Görlitz (51°18′N, 14°42′E). Mücka is located in the center of the large 300 km^2^ forest and pond-rich landscape of the biosphere reserve Oberlausitzer Heide- und Teichlandschaft. This is the most pond-rich area in Germany with about 50% forest and 27.5 km^2^ comprised of ponds, streams and other waters. This area is characterized by riparian forests, moor, pagan, and dune landscapes. Mücka is located 15 km southeast of the Boxberg power plant, a brown coal power plant in the Lusatian brown coal mining district, which, according the 2016 reporting year, had mercury emissions of 512 kg per year (www.thru.de).

The small town of Geislingen an der Steige (75.83 km^2^) is located on the edge of the Middle Swabian Jura in the district of Göppingen, in Baden-Württemberg (48°37′N, 9°49′E). The city is located in the Fils River valley, where they experience occasional flooding. The study area is mainly characterized by agricultural land (52%) and forest (33%). The settlement area covers about 7% and water <1% of the area.

Luxembourg is one of the smallest European countries with an area of 2.586 km^2^. The north of the country (Oesling) is a part of the Ardennes and is characterized by wooded mountains, hills, and deep river valleys. In the south and center of the country is the Gutland region, comprising 68% of the Luxembourg territory. Forests and agricultural areas cover 85% of the total land. Most industry in Luxembourg is found in Gutland. In addition to a large iron mill in Differdange, there are also companies producing chemical goods (such as car tires, plastics (synthetic materials), synthetic fibers, glass, ceramics). An investigation of four fish species from seven rivers (Syrbaach, Sȗre, Our, Wark, Wiltz, Troine, Clerve) in northern Luxembourg (Oesling) showed mercury levels of 10.3 to 534.5 ng g^−1^ wet weight (wt) (Boscher et al. 1996).

### Animal hair sample preparation

This study was performed on the carcasses of adult raccoons from Germany (*n* = 18) and Luxembourg (*n* = 10) and wildcats from Luxembourg (*n* = 15) collected between 2015 and 2016. The wildcats had been killed in traffic and the raccoons were legally hunted.

Samples of hair were collected from the back near the shoulder blades. Approximately 4 g of hair were collected from each animal. Samples were stored in polyvinyl bags until further processing. Hair samples weighing 0.5 g each were washed according to the International Atomic Energy Agency recommended procedure (IAEA 1978) with acetone, then deionised water, and then again with acetone to remove adhered dirt and organic materials. Hair samples were then dried in an oven at 110 °C for 12 h (Curi et al. 2012).

### Hair THg and Se analysis

Total mercury concentrations were determined using atomic absorption spectroscopy (AAS). The assays were run in an AMA 254 mercury analyzer (Altach Ltd, Czech Republic) in accordance with the procedure described by Lanocha et al. (2014). The detection limit for this device is 0.01 ng THg. The analytical procedure was checked by determining THg concentrations in samples of two reference materials: DOLT-4 (Dogfish Liver Certified Reference Material for Trace Metals, Canadian Irradiation Centre, Laval, Quebec; *n* = 3) and 8414 NIST (Bovine Muscle Powder National Institute of Standards and Technology NIST, Canada; *n* = 5). The recovery rates were 99.2% and 108% for DOLT-4 and 8414 NIST, respectively.

Selenium concentrations were measured using spectrofluorimetry. Samples were digested in HNO_3_ at 230 °C for 180 min and in HClO_4_ at 310 °C for 20 min. Then, 9% HCl was added to reduce Se^6+^ to Se^4+^. Selenium was determined by reaction with 2,3-diaminonaphthalene (Sigma Aldrich) under controlled pH conditions (pH 1–2) producing selenodiazole complex, which was then extracted into cyclohexane. More details of the analytical procedures are given by Pilarczyk et al. (2010). Selenium concentration was determined fluorometrically using a RF-5001 PC Shimadzu spectrophotofluorometer. The excitation wavelength was 376 nm and the fluorescence emission wavelength was 518 nm. The detection limit of Se was 0.003 μg g^−1^. The procedure was validated with certified reference material NCS ZC 71001 (bovine liver, China National Analysis Center for Iron and Steel, Beijing, China; *n* = 5). The mean Se concentration was 90% of the reference value. Measured Se and THg concentrations were expressed as mg kg^−1^ dry weight (dw).

### Se:THg molar ratio calculation

Dry weight measurements were converted to molar concentrations by the following formula: molar concentration (nmol g^−1^) = concentration (nmol g^−1^) × 1000/atomic weight (g mol^−1^). Atomic weights of Se and THg are 78.96 g mol^−1^ and 200.59 g mol^−1^, respectively. The calculated Se and THg concentrations were used to calculate Se:THg molar ratio (Kalisinska et al. 2017).

### Statistical analysis

Statistical analyses were performed using Stat Soft Statistica 12.0 and Microsoft Excel 2016. Arithmetic means (AM), standard deviations of the AM (SD), median (Med), and minimum/maximum (ranges) were calculated. Distribution of empirical data on THg and Se concentrations in the hair of the studied animals diverged from the expected normal distribution, as shown by the Kolmogorov–Smirnov test with Lilliefors correction. Therefore, in comparisons of mean values of THg and Se concentration, nonparametric Mann–Whitney U tests were used. In addition, Spearman’s rank correlation coefficients (*R*) were calculated for the relations between THg levels, Se levels, and Se:THg molar ratio in the collected hair, and weight of the animals, as well as between the analyzed elements. Statistical significance was determined at *p* < 0.05.

## Results

The concentrations of THg and Se, and Se:THg molar ratio in the hair of the raccoons and wildcats are presented in Table 1. The total mercury levels were higher in hair from raccoons, but the difference was not statistically significant. The selenium concentrations were higher in the hair of raccoons than of wildcats. The THg concentration in the hair of raccoons from Luxembourg was almost 5× higher than raccoons from Germany. Se concentration was similar in animals from both countries. The molar ratio of Se:THg was ~4× higher in the hair of the raccoons from Germany than those from Luxembourg.

**Table 1:** The total mercury (THg) and selenium (Se) concentrations and Se:THg molar ratio in the hair of raccoons and wildcats from Germany and Luxembourg (AM arithmetic mean, Med median, SD standard deviation, U-Mann–Whitney *U* test, *p*-level of significance, NS non-significant difference, THg and Se concentration are expressed in mg kg^−1^ dw, molar concentration in brackets)

			THg	Se	Se:THg
Raccoons	Total (*n* = 28)	AM ± SD	1.512 ± 1.731 (0.006 ± 0.009)	0.840 ± 0.276 (0.012 ± 0.003)	6.600 ± 5.908
		Med	0.369 (0.002)	0.851 (0.011)	5.324
		Range	0.098–7.461 (0.000–0.037)	0.386–1.498 (0.005–0.019)	0.351–24.160
	Germany (*n* = 18)	AM ± SD	0.483 ± 0.704 (0.002 ± 0.004)	0.802 ± 0.321 (0.013 ± 0.004)	8.936 ± 6.080
		Med	0.221 (0.001)	0.747 (0.009)	7.630
		Range	0.098–2.805 (0.000–0.014)	0.386–1.498 (0.005–0.019)	0.416–24.160
	Luxembourg (*n* = 10)	AM ± SD	2.358 ± 2.353 (0.012 ± 0.012)	0.930 ± 0.147 (0.012 ± 0.002)	2.395 ± 2.076
		Med	1.508 (0.008)	0.922 (0.012)	1.967
		Range	0.394–7.461 (0.002–0.037)	0.674–1.120 (0.009–0.014)	0.351–6.233
Wildcats	Luxembourg (*n* = 15)	AM ± SD	0.685 ± 1.007 (0.003 ± 0.005)	0.678 ± 0.189 (0.009 ± 0.002)	10.979 ± 14.743
		Med	0.273 (0.001)	0.641 (0.008)	4.037
		Range	0.035–3.669 (0.000–0.018)	0.338–1.026 (0.004–0.013)	0.427–49.735
Raccoon vs. wildcats			NS	*U* = 130 *p* < 0.05	NS
Raccoon: Germany vs. Luxembourg			*U* = 17 *p* < 0.001	NS	*U* = 24 *p* < 0.001

Measured concentrations of analyzed elements and Se:THg molar ratio with respect to animal sex are presented in Table 2. The total mercury in hair of female wildcats was ~1.4× higher than in male wildcats, and more than 2× higher in male raccoons than in female raccoons, but these differences were not statistically significant. Se concentration was similar in the hair of male and female wildcats as well as between male and female raccoons.

**Table 2:** The total mercury (THg) and selenium (Se) concentrations and Se:THg molar ratio in hair of wildcat and raccoons according to the gender of animals (AM arithmetic mean, Med median, SD standard deviation, U-Mann–Whitney *U* test, *p*-level of significance, NS non-significant difference; THg and Se concentration are expressed in mg kg^−1^ dw; molar concentration in brackets)

			THg	Se	Se:THg
Raccoon	Male (*n* = 13)	AM ± SD	1.667 ± 2.331 (0.008 ± 0.012)	0.803 ± 0.323 (0.010 ± 0.004)	5.289 ± 5.244
		Med	0.412 (0.002)	0.771 (0.010)	4.831
		Range	0.127–7.461 (0.001–0.037)	0.386–1.498 (0.005–0.019)	0.351–18.974
	Female (*n* = 15)	AM ± SD	0.706 ± 0.812 (0.004 ± 0.004)	0.887 ± 0.232 (0.011 ± 0.003)	7.736 ± 6.385
		Med	0.344 (0.002)	0.877 (0.011)	6.233
		Range	0.098–2.805 (0.000–0.014)	0.459–1.272 (0.006–0.016)	0.416–24.160
	M vs. F	NS	NS	NS	
Wildcat	Male (*n* = 9)	AM ± SD	0.600 ± 0.708 (0.003 ± 0.004)	0.689 ± 0.235 (0.009 ± 0.003)	6.036 ± 4.719
		Med	0.273 (0.001)	0.642 (0.008)	4.037
		Range	0.124–2.196 (0.001–0.011)	0.338–1.026 (0.004–0.013)	1.026–15.480
	Female (*n* = 6)	AM ± SD	0.812 ± 1.416 (0.004 ± 0.007)	0.661 ± 0.105 (0.008 ± 0.001)	18.394 ± 21.518
		Med	0.296 (0.001)	0.647 (0.008)	8.166
		Range	0.035–3.669 (0.000–0.018)	0.509–0.788 (0.006–0.010)	0.427–49.735
	M vs. F	NS	NS	NS	

Significant negative correlation coefficients were found for the relationship between THg and Se:THg molar ratio in wildcats (*R* = −0.97; *p* < 0.05) and raccoons (*R* = −0.93; *p* < 0.05). There were no correlations in either wildcats or raccoons for Se-THg or Se-Se:THg.

## Discussion

Hair is a keratinaceous substance, rich in sulfhydryl-containing amino acids that avidly bind certain trace elements. Mercury and selenium, in particular, have an affinity for the sulfur-rich amino acids in hair, and tend to be found in high concentrations there (Burger et al. 1994). Hair is commonly used to monitor trace element concentrations because it can easily be collected from both live and dead animals (Lord et al. 2002). Moreover, changes in trace elements in skin formations may reflect age-related changes in metabolic profiles across the life span and thus, may also be used to predict risk of age-related chronic diseases (Ambeskovic et al. 2013). Some researchers have found that hair THg levels are correlated with THg concentrations in the kidney and liver (Cumbie 1975; Halbrook et al. 1994); however, reports also exist to the contrary (Lord et al. 2002). Many studies have focused on measuring Hg concentration in the hair of piscivorous mammals such as the mink and otter (Table 3). In wild animal hair Hg occurs mainly as methylmercury and organic mercury comprising 80% and 20%, respectively. There, MeHg is incorporated into the cornified epithelium during hair growth where it is not biologically available, but remains an indicator of previous levels of exposure (Wang et al. 2014).

**Table 3:** Comparison of mercury (Hg) and selenium (Se) concentration in hair of raccoons (*Procyon lotor*), Eurasian otter (*Lutra lutra*), river otter (*Lontra canadensis*), giant otther (*Pteronura brasilensis*), mink (*Neovison vison*), muskrat (*Ondatra zibethicus*), jaguar (*Panthera onca*), cats (*Felis s. catus)*, golden jackal (*Canis aureus*), red fox (*Vulpes vulpes*) and arctic fox (*Vulpes lagopus*) collected in different regions of the world (*n* number of samples, *dw* dry weight, *ww* wet weight, *ad* adults, *imm* immature, *M* male, *F* female)

Species	*n*	Diet	Localization	Hg concentration	Se concentration	References
Terrestrial and omnivorous						
Raccoons (*Procyon lotor*)	10	~44% mammals, 26% fish and frogs, 12% invertebrates, 2% plant material (Drygala et al. 2014, Goszczynski et al. 2000)	Emory River embayment of Watts Bar Reservoir in east Tennessee, USA unexposed to coal fly ash	0.3 mg kg^−1^ ww	2.85 mg kg^−1^ ww	Souza et al. 2013
	10		Exposed to coal fly ash in 2009	0.36 mg kg^−1^ ww	3.25 mg kg^−1^ ww	
	10		Exposed to coal fly ash in 2010	0.47 mg kg^−1^ ww	4.55 mg kg^−1^ ww	
	8		Kesterson Reservoir, California, USA (Se contaminated irrigation drainwater)		28.3 mg kg^−1^ dw	Clark et al. 1989
	4		Volta Wildlife Area, California, USA		0.93 mg kg^−1^ dw	
	14		U.S. Department of Energy’s Savannah River Site Steel Creek delta	1.65 mg kg^−1^ dw		Lord et al. 2002
	18		Upper three runs	1.49 mg kg^−1^ dw		
	20		Pond B	0.89 mg kg^−1^ dw		
	16		Ash basins	1.00 mg kg^−1^ dw		
	24		Offsite	0.65 mg kg^−1^ dw		
	11		South Florida, USA	10.6 mg kg^−1^ ww		Porcella et al. 2004
Semiaquatic and piscivorous						
Eurasian otter (*Lutra lutra*)	36	Seasonally up to 98% fish, 1.2% amphibians (Krawczyk et al. 2011; Sidorovich et al. 1998)	UK	18.75 mg kg^−1^ dw		Mason et al. 1986
River otter (*Lontra canadensis*)	27	Fish, crayfish, frogs, crabs, birds, eggs (Day et al. 2015)	Wisconsin Northern zone	7.05 l µg g^−1^ ww		Strom 2008
	35		Central zone	6.04 l µg g^−1^ ww		
	20		Southern zone	3.40 l µg g^−1^ ww		
	41		South-Central Ontario, Canada	78.6 mg kg^−1^ ww		Evans et al. 2000
	18		Herschel	10674 ng g^−1^ dw		Evans et al. 1998
	9		Harcourt	9202 ng g^−1^ dw		
	12		Cardiff	8866 ng g^−1^ dw		
	13		Wallbridge	9718 ng g^−1^ dw		
	55		Ware Co.	25.55 mg kg^−1^ ww		Halbrook et al. 1994
	36		Echols Co.	22.95 mg kg^−1^ ww		
	34		Piedmont	15.24 mg kg^−1^ ww		
	3		Piedmont	15.9 mg kg^−1^ dw		Cumbie 1975
	6		Lower Coastal Plain of Georgia	37.6 mg kg^−1^ dw		
	33		Central Saskatchewan, Canada	9.68 mg kg^−1^ ww		Wilkie et al. 2018
	71		Ontario, Canada	7.9 mg kg^−1^ dw		Klenavic et al. 2008
	80		Québec, Canada	16.0 mg kg^−1^ dw		
	48		Nova Scotia, Canada	38.0 mg kg^−1^ dw		
Giant otter (*Pteronura brasilensis*)	2	Manly fish, rarely mammals, amphibians, reptiles, birds, crustaceans (Carter and Rosas 1997)	Rio Negro River, Brasil	2.94–3.68 mg kg^−1^ dw		Dias Fonseca et al. 2005
Mink (*Neovison vison*)	1	Seasonally up to 62% fish, 56% mammals, 4-16% birds (Barrat et al. 2010, Bartoszewicz and Zalewski 2003)	South Saskatchewan River, Canada	34.9 mg kg^−1^ dw		Wobeser and Swift 1976
	19		South-Central Ontario, Canada	75.0 mg kg^−1^ ww		Evans et al. 2000
	25		Ontario, Canada	7.4 mg kg^−1^ dw		Klenavic et al. 2008
	54		Québec, Canada	24.0 mg kg^−1^ dw		
	65		Nova Scotia, Canada	24.0 mg kg^−1^ dw		
	1		U.S. Department of Energy Oak Ridge Reservation, USA	104.0 mg kg^−1^ dw		Stevens et al. 1997
	3		Bear Creek	11.0 mg kg^−1^ dw		
	1		White Oak Creek	8.8 mg kg^−1^ dw		
	7		Reference sites	5.15 mg kg^−1^ dw		
	5		Piedmont	10.7 mg kg^−1^ dw		Cumbie 1975
	2		Lower Coastal Plain of Georgia			
Terrestrial and carnivorous						
Jaguar (*Panthera onca*)	9	87% mammals, 9.8% reptiles, 2.8 birds (Garla et al. 2001)	Pantanal, Centralwestern Brazil; gold mining	673 mg kg^−1^ dw		May Júnior et al. 2017
			References area	29.7 mg kg^−1^ dw		
Cats (*Felis s. catus)*		Fish	Minamata District, Japan	45.9 mg kg^−1^ dw		Kitamura 1968
Golden jackal (*Canis aureus*)	21	42.7% mammals, 12.0% birds, 27.3% plants, 18.0% insects (Giannatos et al. 2010)	Mazandaran Province, Iran	178.3 ng g^−1^ dw		Malvandi et al. 2010
Red fox (*Vulpes vulpes*)	200	Seasonally up to 60% small mammals, 21% carrion, ~ 20% birds, up to 26% plant material (Goldyn et al. 2003)	Kuskokwim River, Alaska	2.58 mg kg^−1^ dw		Dainowski et al. 2015
Arctic fox (*Vulpes lagopus*)		Small rodents 54.7%, reindeer 13.3%, hare 12%, birds 9.8%, vegetation 9.5%, other 0.6% (Strand et al. 2000)	Mednyi Island, Russia	10.42 mg kg^−1^ dw		Bocharova et al. 2013
	7 (M, imm.)		Iceland	3.22 mg kg^−1^ ww		Treu et al. 2018
	5 (F, imm.)			5.09 mg kg^−1^ ww		
	15 (M, ad.)			10.15 mg kg^−1^ ww		
	8 (F, ad.)			9.70 mg kg^−1^ ww		

When we start to analyze the results of individual species in the table and their several degrees of contamination with heavy metals we can recognize distinct differences as well relationships. One of them is variance in Hg level between raccoon, which is an omnivorous species and semiaquatic species, like otter or grand otter. The results show clearly, that the bigger share of fish and water animals in the food is connected to higher percentage of Hg deposition in tissues of several predators. Also the results measured by cats in Japan preying on fish confirm the assumption. Raccoon, as omnivorous species in lower risk to be highly contaminated, but also this depends from the specific place. And so we can observe, that in areas with ash basins and coal fly ash even terrestrial individuals or animals hunting in high percentage on mammals can be laden with heavy metals. Similar results were observed in jaguar in Pantanal, what stays in connection to gold mining. When we also consider the results from Mednyi Island we can see several examples of contamination with Hg. The island belongs to Kamchatka, which main industries is although fishing and forestry, but also coal and other raw materials are extracted. It could be an explanation for the evidence of partial contamination in foxes, which avoid fish but despite this show in their foraging similarities to raccoons, and proofs that in Hg contaminated areas also terrestrial organism can be higher laden with this element. Some percentage of Hg contamination we can see also in Iceland. Although Iceland is not very industry country, but carnivores living there prey on birds eating fish and on this way can Hg contained in water get to into their bodies. Island lays in the straight line about 1200 km from Greenland, where iron, uranium, aluminium, nickel, platinum, tungsten, titanium and copper are mined (Nordic Labour Journal 2011; Guidelines for the Evaluation of Petroleum Reserves and Resources 2013).

Due to lack of Se results by other species we can observe similar relationships on example of raccoon. Also in this case higher tissues contamination can be connected to higher environmental and water pollution, like in Kesterson Reservoir, California,. Due to several investigations raccoons prey frequently in ¼ on fish, in which bodies heavy metals can be deposited

Smaller mammals, such as mink, cats, otters, and raccoons appear to be more resistant to Hg than larger animals, likely due to differences in the rate of metabolism and a higher rate of detoxification (Eisler 2006; Lanocha et al. 2014). It has been estimated that THg concentrations ranging from 1 to 5 mg kg^−1^ in the hair of piscivorous mammals represent normal background levels (Sheffy and St. Amant 1982). Based on a literature review, the US Fish and Wildlife Service proposed that a level of 1.1 mg kg^−1^ ww (5.5 mg kg^−1^ dw) in hair and organs with central roles in detoxification “should be considered as presumptive evidence of an environmental mercury problem” (Eisler 2000; Lanocha et al. 2014). Moreover, hair THg levels of 30 mg kg^−1^ have been identified as the lowest observed adverse effect level (LOAEL) for terrestrial mammalian wildlife (Basu et al. 2007; Crowley et al. 2018; Evers et al. 2007).

Ecotoxicological studies with raccoons, conducted mainly in North America, have examined mainly the liver, kidney, and, less frequently, the hair (Lanocha et al. 2014; Lord et al. 2002; Porcella et al. 2004; Souza et al. 2013). Literature shows hair THg levels in piscivorous mammals, such as the raccoon, ranging from ~0.3 to ~13 mg kg^−1^ dw (Table 3). In this study, average THg concentration in raccoons was 1.5 mg kg^−1^ dw, similar to that reported by Lord et al. (2002) in the hair of raccoons from the U.S. Department of Energy’s Savannah River Site Steel Creek delta, an area heavily polluted with Hg compounds. In our study, the highest THg levels (~7.5 mg kg^−1^ dw) were found in raccoons from the forested and agricultural areas near Junglinster, Luxembourg. Increased concentrations of various elements in the fields, including organic forms of Hg, may possibly lead to changes in animal organ function, thus increasing tissue Hg levels (Strickman and Mitchell 2017).

We have not found existing reports on levels of trace elements in the hair of European wildcats. Measured THg in felids in non-contaminated areas does not exceed 30 mg kg^−1^ dw (May Júnior et al. 2017), while in contaminated areas it ranges from 46 to 673 mg kg^−1^ dw (Kitamura 1968; May Júnior et al. 2017). In this study, average THg concentration in European wildcats was 0.73 mg kg^−1^ dw, about 40x lower than in the hair of jaguars from a reference area in Brazil. The highest observed THg content in feline hair was recorded in jaguars from a region affected by gold mining and Hg contamination (May Júnior et al. 2017).

Although Se plays an important role in hair growth, little is known about its levels in skin formations. Hair, similar to bone tissue, may reflect the long-term Se level in the organism. Selenium-deficient rat pups display a slower growth rate and sparse hair growth (Bates et al. 2000). Sengupta et al. (2010) credit selenoproteins for the protective roles of selenium in skin and establish that deficiencies in selenoproteins beget most skin abnormalities associated with selenium deficiency. In raccoon hair, Se may be excreted at a higher rate than in other animals (Souza et al. 2013). In our study, hair median Se levels in the raccoon and European wildcat were 0.851 and 0.641 mg kg^−1^ dw, respectively, a statistically significant difference. Higher Se concentration in raccoon hair was likely a result of higher absorption of plant Se, resulting from a diet containing a significant amount of plants.

Selenium concentration similar to that found in the present study was noted by Clark et al. (1989) in the hair of raccoons from and area known to have a low Se concentration (Volta Wildlife Area, California, USA). They found much higher Se levels (28.3 mg kg^−1^ dw) in raccoon hair from an Se-contaminated irrigation drain water zone (Kesterson Reservoir, California, USA). The lower Se concentrations in the raccoons and wildcats in our study may result from naturally low soil Se levels in Germany and Luxembourg (Reimann et al. 2014).

Levels of toxic elements, including Hg, in the hair, may be affected by biotic and abiotic factors including age, sex, sampling season, and feeding strategy (Treu et al. 2018). In the present study, no differences were found in THg and Se concentrations between male and female raccoons or European wildcats. This confirms the results of previous studies showing no sex-dependence of Hg concentration in the hair of cats (Sakai et al. 1995) and mink (Stevens et al. 1997). In contrast, a sex-related difference was observed in previous studies on the raccoon (Porcella et al. 2004) and panther (Newman et al. 2005).

Concentrations of elements in hair also depend on the physiological, hormonal, and individual characteristics of the species. The binding of trace elements to hair-forming proteins depends on the amount of protein, carbohydrates, and lipids supplied, as well as the penetration of these elements into cells through diet and their use in metabolic processes (Baumgartner et al. 1981). The raccoon diet consists mainly of a variety of molluscs, fish, amphibians, insects, birds, vegetables, and fruits, with some carrion and garbage (Bartoszewicz et al. 2008). The European wildcat is a medium-sized carnivore, preying on rodents, insectivores, lagomorphs, reptiles, insects, and eating some plants (Lozano et al. 2006; Moleon and Gil-Sanchez 2003; Sarmento 1996). These dietary differences are likely responsible for the observed discrepancy between the concentrations of the investigated elements in the hair of these two animals.

Many reports stress the value of not only determining concentrations of Hg and Se, but also examining their molar ratio in wildlife. Tissue Se:THg molar ratios of <1 may be connected with a potential increase in Hg toxicity. In warm blooded animals (specifically marine species), an important detoxification mechanism relies on the antagonistic impact of Se on Hg. Kalisinska et al. (2017) suggested that piscivores in Se-deficient areas are more exposed to Hg than fish and omnivores, further exacerbating the effect of this deficiency. Moreover, in the raccoons from Warta Mouth National Park (Poland), an area with moderate Hg pollution, it was found that Se:THg molar ratios in muscle spanned a wide range, from 0.73 to ~66. Data on Hg, Se, and Se:THg ratios in terrestrial mammals are limited. In the present study, Se:THg ratio in raccoon hair (Luxembourg and Germany) ranged from 0.351 to 24.16, and in wildcats (Luxembourg) it ranged from 0.427 to 41.11. In some individual specimens, the hair Se:THg molar ratio was <1, which could enhance THg toxicity. However, in most cases these values exceeded 1, thus likely effectively protecting against the adverse effects of Hg.

Some authors suggest that the molar ratio of Se:Hg in warm-blooded vertebrate tissues depends on the trophic group and species of the animal, the type of tissue being examined, and the area from which the animals came (Burger and Gochfeld 2013). In the presented study, statistically significant differences were found in Se:THg ratio between raccoon hair from Germany and from Luxembourg.

The highest exposure to Hg is recorded in piscivorous animals, with Se:THg molar ratio in the organs of otters and minks not exceeding 1 (Kalisinska et al. 2017; Wren 1984). Our study suggests that omnivorous raccoons and predatory wildcats are less exposed to the toxic effects of Hg, likely due to a smaller share of fish in their diet, the main source of mercury for these animals, the rate of Hg absorption, and considerable intake of easily digestible Se from plants (50%) or meat (35%). It should be noted however, that regardless of species, Hg-polluted and Se-deficient areas may influence the Se:Hg ratio, possibly leading to toxic effects in wild mammals.

## Conclusions

Analysis of the concentration of THg, Se and Se:THg molar ratio in the hair of raccoons (*Procyon lotor)* and European wildcats (*Felis s. silvestris*) from Germany and Luxembourg showed that: THg concentration was almost 5× higher in raccoons from Luxembourg than from Germany. Selenium concentration was significantly higher in raccoons than in wildcats. Sex does not influence the concentration of THg and Se in the hair of raccoons or European wildcats. Average Se:THg ratio was >1 in studied animals, which indicates that omnivorous raccoons and predatory wildcats are less exposed to the toxic effects of THg than piscivorous animals.

## References

[CR1] Ahsan U, Kamran Z, Raza I (2014). Role of selenium in male reproduction—a review. Anim Reprod Sci.

[CR2] Ambeskovic M, Fuchs E, Beaumier P (2013). Hair trace elementary profiles in aging rodents and primates: links to altered cell homeodynamics and disease. Biogerontology.

[CR3] Bartoszewicz M, Okarma H, Zalewski A (2008). Ecology of the raccoon (*Procyon lotor*) from western Poland. Ann Zool Fennici.

[CR4] Barrat J, Richomme C, Moinet M (2010). The accidental release of exotic species from breeding colonies and zoological collections. Rev Sci Tech Int Epizoot.

[CR5] Bartoszewicz M, Zalewski A (2003). American mink, *Mustela vison* diet and predation on waterfowl in the Słońsk *R*eserve, western Poland. Folia Zool.

[CR6] Basu N, Scheuhammer AM, Bursian SJ (2007). Mink as a sentinel species in environmental health. Environ Res.

[CR7] Bates JM, Spate VL, Morris JS (2000). Effects of selenium deficiency on tissue selenium content, deiodinase activity, and thyroid hormone economy in the rat during development. Endocrinology.

[CR8] Baumgartner A, Jones P, Black C (1981). Detection of phencyclidine in hair. J Forensic Sci.

[CR9] Beau F, Bustamante P, Michaud B (2019). Environmental causes and reproductive correlates of mercury contamination in European pond turtles (*Emys orbicularis*). Environ Res.

[CR10] Bigler WJ, Jenkins JH, Cumbie PM (1975). Wildlife and environmental health: raccoons as indicators of zoonoses and pollutants in Southeastern United States. J Am Vet Med Assoc.

[CR11] Biró Z, Szemethy L, Heltai M (2004). Home range sizes of wildcats (*Felis silvestris*) and feral domestic cats (*Felis silvestris* f. catus) in a hilly region of Hungary. Mamm Biol.

[CR12] Bjerregaard P, Fjordside S, Hansen MG (2011). Dietary selenium reduces retention of methyl mercury in freshwater fish. Environ Sci Technol.

[CR13] Bjørklund G, Aaseth J, Ajsuvakova OP (2017). Molecular interaction between mercury and selenium in neurotoxicity. Coord Chem Rev.

[CR14] Bocharova N, Treu G, Czirják GÁ (2013). Correlates between feeding ecology and mercury levels in historical and modern arctic foxes (*Vulpes lagopus*). PLoS ONE.

[CR15] Boscher A, Gobert S, Guignard C et al. (1996) Biosphärenreservates Oberlausitzer Heide—und Teichlandschaft. Biosphärenreservatsplan Teil 1. Grundlagen für Schutz, Pflege und Entwicklung, Mücka

[CR16] Burger J, Gochfeld M (2013). Selenium/mercury molar ratios in freshwater, marine, and commercial fish from the USA: variation, risk, and health management. Rev Environ Health.

[CR17] Burger J, Marquez M, Gochfeld M (1994). Heavy metals in the hair of opossum from Palo Verde, Costa Rica. Arch Environ Contam Toxicol.

[CR18] Carter SK, Rosas FC (1997). Biology and conservation of the giant otter *Pteronura brasiliensis*. Mamm Rev.

[CR19] Castellini J, Rea LD, Lieske CL (2012). Mercury concentrations in hair from neonatal and juvenile Steller Sea Lions (*Eumetopias jubatus*): implications based on age and region in this northern Pacific marine sentinel piscivore. EcoHealth.

[CR20] Castoldi AF, Coccini T, Ceccatelli S (2001). Neurotoxicity and molecular effects of methylmercury. Brain Res Bull.

[CR21] Cho JM, Yang HR (2018). Hair mineral and trace element contents as reliable markers of nutritional status compared to serum levels of these elements in children newly diagnosed with inflammatory bowel disease. Biol Trace Elem Res.

[CR22] Clark DR, Ogasawara PA, Smith GJ (1989). Selenium accumulation by raccoons exposed to irrigation drainwater at Kesterson National Wildlife Refuge, California, 1986. Arch Environ Contam Toxicol.

[CR23] Crowley SM, Hodder DP, Johnson CJ (2018). Wildlife health indicators and mercury exposure: a case study of river otters (*Lontra canadensis*) in central British Columbia, Canada. Ecol Indic.

[CR24] Cumbie PM (1975). Mercury in hair of bobcats and raccoons. J Wildl Manag.

[CR25] Curi NH, Brait CH, Antoniosi Filho NR (2012). Heavy metals in hair of wild canids from the Brazilian Cerrado. Biol Trace Elem Res.

[CR26] Cuvin-Aralar ML, Furness RW (1991). Mercury and selenium interaction: a review. Ecotoxicol Environ Saf.

[CR27] Dainowski BH, Duffy LK, McIntyre J (2015). Hair and bone as predictors of tissular mercury concentration in the western Alaska red fox, *Vulpes vulpes*. Sci Total Environ.

[CR28] Davis LE, Kornfeld M, Mooney HS (1994). Methylmercury poisoning: long-term clinical, radiological, toxicological, and pathological studies of an affected family. Ann Neurol.

[CR29] Day CC, Westover MD, McMillan BR (2015). Seasonal diet of the northern river otter (L*ontra canadensis)*: what drives prey selection?. Can J Zool.

[CR30] Dias Fonseca FR, Malm O, Francine Waldemarin H (2005). Mercury levels in tissues of Giant otters (P*teronura brasiliensis)* from the Rio Negro, Pantanal, Brazil. Environ Res.

[CR31] Drygala F, Wernerb U, Zoller H (2014). Diet composition of the invasive raccoon dog (N*yctereutes procyonoides)*. Hystrix.

[CR32] Eisler R (2000) Handbook of chemical assessment: health hazards to humans, plants and animals, vol I. Lewis, Washington, DC, USA

[CR33] Eisler R (2006). Mercury hazards to living organisms.

[CR34] Evans RD, Addison EM, Villeneuve JY (1998). An examination of spatial variation in mercury concentrations in otter (*Lutra canadensis*) in south-central Ontario. Sci Total Environ.

[CR35] Evans RD, Addison EM, Villeneuve JY (2000). Distribution of inorganic and methylmercury among tissues in mink (*Mustela vison*) and otter (*Lutra canadensis*). Environ Res Sect A.

[CR36] Evers DC, Han YJ, Driscoll CT (2007). Biological mercury hotspots in the Northeastern United State and Southeastern Canada. BioScience.

[CR37] Fordyce FM (2013) Selenium deficiency and toxicity in the environment. Essent of Med Geol 375–416

[CR38] Garla RC, Setz EF, Gobbi N (2001). Jaguar (*Panthera onca*) food habits in Atlantic Rain Forest of southeastern Brazil. Biotropica.

[CR39] Giannatos G, Karypidou A, Legakis A, Polymeni R (2010). Golden jackal (Canis aureus L.) diet in Southern Greece. Mamm Biol.

[CR40] Goldyn B, Hromada M, Surmacki A, Tryjaowski P (2003). Habitat use and diet of the red fox *Vulpes vulpes* in an agricultural landscape in Poland. Z Jagdwiss.

[CR41] Goszczynski J, Jedrzejewska B, Jedrzejewski W (2000). Diet composition of badgers (*Meles meles)* in a pristine forest and rural habitats of Poland compared to other European populations. J Zool Lond.

[CR42] Górski K, Kondracki S, Saba L (2018). Selenium concentration in soil, and in the feed and hair coat of Polish Holstein-Friesian cows administered a mineral mixture. Indian J Anim Sci.

[CR43] Greenland’s red hot labour market, Nordic Labour Journal, 2011

[CR44] Society of petroleum engineers (2013) Guidelines for the evaluation of petroleum reserves and resources. Society of petroleum engineers

[CR45] Graeme KA, Pollack CV (1998). Heavy metal toxicity, Part I: arsenic and mercury. J Emerg Med.

[CR46] Halbrook RS, Jenkins JH, Bush PB (1994). Sublethal concentrations of mercury in river otters: monitoring environmental contamination. Arch Environ Contam Toxicol.

[CR47] Herbert GB, Peterle TJ (1990). Heavy metal and organochlorine compound concentrations in tissues of raccoons from east-central Michigan. Bull Environ Contam Toxicol.

[CR48] Herrmann M, Kitchener A, Meinig H et al. (2007) *Felis silvestris*. The IUCN red list of threatened species 2007:e.T60354712A112955994, https://www.iucnredlist.org/species/60354712/112955994

[CR49] Izquierdo A, Casas C, Herrero E (2010). Selenite-induced cell death in *Saccharomyces cerevisiae*: protective role of glutaredoxins. Microbiology.

[CR50] Kalisińska E, Kalisińska E (2019). Endothermic animals as biomonitors of terrestrial environments. Mammals and birds as bioindicators of trace element contaminations in terrestrial environments. An ecotoxicological assessment of the northern hemisphere.

[CR51] Kalisinska E, Lanocha-Arendarczyk N, Kosik-Bogacka D (2017). Muscle mercury and selenium in fishes and semiaquatic mammals from a selenium-deficient area. Ecotoxicol Environ Saf.

[CR52] Khan WA, Wang F (2010). Chemical demethylation of methylmercury by selenoamino acids. Chem Res Toxicol.

[CR53] Kitamura S (1968) Determination of mercury content in bodies of inhabitants, cats, fishes, and shells in Minamata District and in the mud of Minamata Bay. In: Kitsuna M (ed) Minamata disease. Kumamoto University Press, Kumamoto, Japan, p 257–266

[CR54] Klar N, Fernández N, Kramer–Schadt S (2008). Habitat selection models for European wildcat conservation. Biol Conserv.

[CR55] Klenavic K, Champoux L, Mike O (2008). Mercury concentrations in wild mink (*Mustela vison*) and river otters (*Lontra canadensis*) collected from eastern and Atlantic Canada: relationship to age and parasitism. Environ Pollut.

[CR56] Krawczyk AJ, Skierczyński M, Tryjanowski P (2011). Diet of the Eurasian otter *Lutra lutra* on small watercourses in western Poland. Mammalia.

[CR57] Lailson-Brito J, Cruz R, Dorneles PR (2012). Mercury-selenium relationships in liver of Guiana dolphin: the possible role of Kupffer cells in the detoxification process by tiemannite formation. PLoS ONE.

[CR58] Lanocha N, Kalisinska E, Kosik-Bogacka DI (2014). Mercury levels in raccoons (*Procyon lotor*) from the Warta Mouth National Park, northwestern Poland. Biol Trace Elem Res.

[CR59] Lieske CL, Moses SK, Castellini J (2011). Toxicokinetics of mercury in blood compartments and hair of fish-fed sled dogs. Acta Vet Scand.

[CR60] Lord CG, Gaines KF, Boring CS (2002). Raccoon (*Procyon lotor*) as a bioindicator of mercury contamination at the U.S. Department of Energy’s Savannah River Site. Arch Environ Contam Toxicol.

[CR61] Lozano J, Moleo M, Virgo E (2006). Biogeographical patterns in the diet of the wildcat, *Felis silvestris* Schreber, in Eurasia: factors affecting the trophic diversity. J Biogeogr.

[CR62] Malvandi H, Ghasempouri SM, Esmaili-Sari A (2010). Evaluation of the suitability of application of golden jackal (*Canis aureus*) hair as a noninvasive technique for determination of body burden mercury. Ecotoxicology.

[CR63] Mao J, Pop VJ, Bath SC (2016). Effect of low-dose selenium on thyroid autoimmunity and thyroid function in UK pregnant women with mild-to-moderate iodine deficiency. Eur J Nutr.

[CR64] Mason CF, Last NI, Macdonald SM (1986). Mercury, cadmium, and lead in British otters. Bull Environ Contam Toxicol.

[CR65] May Júnior JA, Quigley H, Hoogesteijn R (2017). Mercury content in the fur of jaguars (*Panthera onca*) from two areas under different levels of gold mining impact in the Brazilian Pantanal. Acad Bras Cienc.

[CR66] Moleon M, Gil-Sanchez JM (2003). Food habits of the wildcat (*Felis silvestris*) inapeculiar habitat: the Mediterranean high mountain. J Zool Lond.

[CR67] Newman J, Zillioux E, Rich E (2005). Historical and other patterns of monomethyl and inorganic mercury in the Florida panther (*Puma concolor coryi*). Arch Environ Contam Toxicol.

[CR68] Nowak R (1997) Walker’s Mammals of the World. The Johns Hopkins University Press, Baltimore. http://www.press.jhu.edu/books/walkers_mammals_of_the_world/carnivora/carnivora.felidae.felis.html. Accessed 12 Mar 2004

[CR69] Ohlendorf Harry, Heinz Gary (2011). Selenium in Birds. Environmental Contaminants in Biota.

[CR71] Pierpaoli M, Biro ZS, Herrmann M (2003). Genetic distinction of wildcat (*Felis silvestris*) populations in Europe, and hybridization with domestic cats in Hungary. Mol Ecol.

[CR72] Pilarczyk Bogumiła, Tomza-Marciniak Agnieszka, Pilarczyk Renata, Marciniak Andrzej, Bąkowska Małgorzata, Nowakowska Ewa (2019). Selenium, Se. Mammals and Birds as Bioindicators of Trace Element Contaminations in Terrestrial Environments.

[CR73] Pilarczyk B, Tomza-Marciniak A, Mituniewicz-Małek A (2010). Selenium content in selected products of animal origin and estimation of the degree of cover daily Se requirement in Poland. Int J Food Sci Technol.

[CR74] Porcella DB, Zillioux EJ, Grieb TM (2004). Retrospective study of mercury in raccoons (P*rocyon lotor)* in south Florida. Ecotoxicology.

[CR75] Qazi IH, Angel C, Yang H (2018). Selenium, selenoproteins, and female reproduction: a review. Molecules.

[CR102] Raymond LJ, Ralston NV (2004) Mercury: selenium interactions and health implications. SMDJ Seychelles Med. Dental J 17:72–77 10.1016/j.neuro.2020.09.020 35587137

[CR76] Rea LD, Correa L, Castellini J (2013). Maternal Steller sea lion diets elevate fetal mercury concentrations in an area of population decline. Sci Total Environ.

[CR77] Reimann C, Birke M, Demetriades A, Filzmoser P, O’Connor P (eds) (2014) Chemistry of Europe’s agricultural soils—Part B: general background information and further analysis of the GEMAS data set. Geologisches Jahrbuch (Reihe B 103), Schweizerbarth, Hannover

[CR78] Rhymer JM, Simberloff D (1996). Extinction by hybridization and introgression. Annu Rev Ecol Evol Syst.

[CR79] Roman M, Jitaru P, Barbante C (2014). Selenium biochemistry and its role for human health. Metallomics.

[CR80] Sakai T, Ito M, Aoki H (1995). Hair mercury concentrations in cats and dogs in central Japan. Br Vet J.

[CR81] Sarmento P (1996). Feeding ecology of the European wildcat *Felis silvestris* in Portugal. Acta Theriol.

[CR82] Scheuhammer AM, Meyer MW, Sandheinrich MB (2007). Effects of environmental methylmercury on health of wild birds, mammals, and fish. Ambio.

[CR83] Sengupta A, Lichti UF, Carlson BA (2010). Selenoproteins are essential for proper keratinocyte function and skin development. PLoS ONE.

[CR84] Sheffy TB, St Amant JR (1982). Mercury burdens in furbearers in Wisconsin. J Wildl Manag.

[CR85] Sidorovich V., Kruuk H., Macdonald D. W., Maran T. (1998). Diets of semi-aquatic carnivores in northern Belarus, with implications for population changes. Behaviour and Ecology of Riparian Mammals.

[CR86] Silva EF, Missio D, Martinez CS (2019). Mercury at environmental relevant levels affects spermatozoa function and fertility capacity in bovine sperm. J Toxicol Environ Health Part A.

[CR87] Stevens RT, Ashwood TL, Sleeman JM (1997). Mercury in hair of muskrats (*Ondatra zibethicus*) and mink (*Mustela vison*) from the U. S. Department of Energy Oak Ridge Reservation. Bull Environ Contam Toxicol.

[CR88] Soares de Campos M, Sarkis JE, Müller RC, Brabo E, Santos E (2002). Correlation between mercury and selenium concentrations in Indian hair from Rondônia State, Amazon region, Brazil. Sci Total Environ.

[CR89] Souza MJ, Ramsay EC, Donnell RL (2013). Metal accumulation and health effects in raccoons (*Procyon lotor*) associated with coal fly ash exposure. Arch Environ Contam Toxicol.

[CR90] Sahl P, Artois M (1994) Status and conservation of the wildcat (*Felis silvestris*) in Europe and around the Mediterranean rim. Nature and Environment Series, No 69. Council of Europe Press, Strasbourg, p 78

[CR91] Strand O, Landa A, Linnell JD, Zimmermann B, Skogland T (2000). Social organization and parental behaviour in arctic foxes, *Alopex lagopus*. J Mammal.

[CR92] Strickman RJ, Mitchell CP (2017). Accumulation and translocation of methylmercury and inorganic mercury in Oryza sativa: An enriched isotope tracer study. Sci Total Environ.

[CR93] Strom SM (2008). Total mercury and methylmercury residues in river otters (*Lutra canadensis*) from Wisconsin. Arch Environ Contam Toxicol.

[CR94] Stubbe M (1993) *Procyon lotor* (Linnaeus, 1758). In: Stubbe M, Krapp F (eds), Handbuch des Saugetiere Europas, vol 5/I. Akademische Verlagsgesellschaft, Wiesbaden, p 331–364

[CR95] Thomson CD (2004). Assessment of requirements for selenium and adequacy of selenium status: a review. Eur J Clin Nutr.

[CR96] Treu G, Krone O, Unnsteinsdóttir ER (2018). Correlations between hair and tissue mercury concentrations in Icelandic arctic foxes (*Vulpes lagopus*). Sci Total Environ.

[CR97] Wang W, Evans RD, Hickie BE (2014). Methylmercury accumulation and elimination in mink (*Neovison vison*) hair and blood: results of a controlled feeding experiment using stable isotope tracers. Environ Toxicol Chem.

[CR98] Wiener JG, Krabbenhoft DP, Heinz GH et al. (2003) Ecotoxicology of mercury. In: Hoffman DJ, Rattner BA, Burton GA, Cairns J (eds) Handbook of ecotoxicology. Lewis, Boca Raton, p 409–463

[CR99] Wilkie SC, Espie RH, Basu N (2018). Trapped river otters (*Lontra canadensis)* from central Saskatchewan differ in total and organic mercury concentrations by sex and geographic location. FACETS.

[CR100] Wobeser G, Swift M (1976). Mercury poisoning in a wild mink. J Wildl Dis.

[CR101] Wren CD (1984). Distribution of metals in tissues of beaver, raccoon and otter from Ontario, Canada. Sci Total Environ.

